# Attitudes of medical students to electroconvulsive therapy

**DOI:** 10.1192/bjo.2021.97

**Published:** 2021-06-18

**Authors:** Patrick Clements, Aidan Turkington

**Affiliations:** Belfast Health & Social Care Trust

## Abstract

**Aims:**

This study explores the different attitudes among fourth year medical students in Queen's University Belfast to Electroconvulsive Therapy (ECT) and investigates whether these are influenced by teaching and exposure to ECT during their undergraduate psychiatry placement. In particular we sought to determine firstly, correlates of baseline attitudes to ECT and secondly, whether specific forms of ECT teaching improved attitudes to ECT during their placement.

**Method:**

This study was conducted in Queen's University Belfast and agreed with their ethics committee. Participants completed a questionnaire at the beginning of their psychiatry placement and another questionnaire in the second half of their placement. The first questionnaire captured background information and baseline attitudes. The second questionnaire recorded the educational and clinical experience gained on ECT during placement (for example lectures, tutorials, informal teaching, observing ECT and interacting with ECT patients), in addition to attitudes to ECT at this timepoint. Attitudes to ECT were assessed on a 5-point Likert scale. A positive attitude to ECT was defined as scoring agree/strongly agree on a 5-point Likert scale to the statement “I would recommend ECT for a patient if clinically indicated”.

**Result:**

187 students were interviewed at both time points. At the outset of the psychiatry placement 66% of students reported a positive attitude to ECT. Positive attitude was associated with age: 72% of students under 24 had a positive attitude to ECT vs 58% of students over 24 (χ2 = 3.5; P < 0.05). Of students who had previously attended a lecture on ECT (n = 117) 83% had a positive attitude to ECT vs 42% of those who had not previously attended a lecture (χ2 = 33.5; P < 0.001).

Attitudes to ECT significantly improved during the placement (66% vs 94% positive; t = 7.97; P < 0.001). Students who attended a lecture on ECT during the psychiatry placement were more likely to have a positive shift in attitude (67% vs 49%; F = 6.0; P = 0.01). No other specific teaching modality was associated with a positive shift in attitude.

**Conclusion:**

We conclude that undertaking a Psychiatry placement and particularly having a lecture on ECT significantly improves attitudes of medical students to ECT. It is therefore important that lectures on ECT are included in the medical undergraduate curriculum to allow students to be accurately informed about this essential treatment for a number of psychiatric disorders.

